# Mitigation of soil water stress by moderately deep sowing and exogenous application of glucosinolates during the early seedling stage in rapeseed

**DOI:** 10.3389/fpls.2026.1752750

**Published:** 2026-03-06

**Authors:** Chenyang Bai, Yizhong Lei, Maria Batool, Ali Mahmoud El-Badri, Ying Chang, Jie Kuai, Bo Wang, Jie Zhao, Zhenghua Xu, Sumera Anwar, Graham John King, Jing Wang, Guangsheng Zhou

**Affiliations:** 1 Ministry of Agriculture (MOA) Key Laboratory of Crop Ecophysiology and Farming System in the Middle Reaches of the Yangtze River, College of Plant Science & Technology, Huazhong Agricultural University, Wuhan, China; 2 Field Crops Research Institute, Agricultural Research Center (ARC), Giza, Egypt; 3 Department of Botany, Government College Women University, Faisalabad, Pakistan; 4 Southern Cross Plant Science, Southern Cross University, Lismore, NSW, Australia

**Keywords:** abiotic stress, canola, crop establishment, seedling vigor, sowing depth

## Abstract

**Introduction:**

Determining the optimal sowing depth suitable for different water conditions is a key agronomic factor for crop establishment and yield potential. This study aimed to identify the optimal sowing depth for rapeseed that maximizes seedling vigor under varying water conditions.

**Methods:**

Seedling emergence and plant growth were evaluated under four water conditions (variable moisture, drought, normal water, and waterlogged) at different sowing depths (1–5 cm). Meanwhile, the hypothesis that seedling vigor under deep sowing conditions could be improved by exogenous application of glucosinolates (GS) was tested.

**Results:**

Results indicated that the highest seedling emergence percentage (EP) was observed at 3 cm, representing increases of 123.2% (variable moisture), 100% (drought), and 11.1% (normal water) compared with 1 cm. Under waterlogged stress, seedling EP showed no significant differences between 1 and 3 cm for 50% of the 16 cultivars. Moreover, seedling EP was significantly improved at 3 cm after seed priming with GS compared with 1 cm, with increases of 46.4% (drought) and 63.0% (waterlogged), whereas no significant differences were observed under normal water conditions. Furthermore, plant phenotypic performance indices were higher at 3 cm with GS treatment than at 1 cm across all water conditions.

**Discussion:**

Collectively, a sowing depth of 3 cm combined with exogenous application of GS not only promoted seedling emergence but also benefited subsequent plant growth in direct-sown rapeseed. These results provide practical insights for ensuring reliable seedling establishment in rapeseed.

## 1 Introduction

Rapeseed/canola (*Brassica napus* L.) is an important oil crop grown in temperate cereal rotations, with a global production area covering approximately 37.1 × 10^6^ ha. Of this, 7 × 10^6^ ha are cultivated in China (FAO, 2020). Soil water balance and water availability play a decisive role in crop growth (Forcella et al., 2000; Yi et al., 2022). Both excess and deficit water lead to difficulties in seedling emergence, poor seedling uniformity, and large fluctuations in plant density, which hinder optimal canopy establishment and result in inadequate harvestable yields. However, soil water deficit has been increasing in major temperate rapeseed production regions due to vegetation changes and global warming (Chai et al., 2025). Indeed, over the past 20 years, long-term drought has become a semipermanent climate in Europe (Christidis and Stott, 2021; Ionita et al., 2022). The Yangtze River Basin (YRB) accounts for about 90% of rapeseed area in China, 80% of which is direct-seeded semi-winter type. Meteorological data from the past 10 years from the National Meteorological Information Center (China Meteorological Administration) also show frequent drought stress during the sowing period (September–October). Over the next 30 years, many regions in China are predicted to experience more frequent, severe, and prolonged droughts (Zeng et al., 2021). Rapeseed seeds are small and possess epigeal cotyledons, with recommendations for a suitable field sowing depth of about 1 cm (Harker et al., 2012). However, topsoil water content is subject to considerable fluctuations, which exacerbate drought stress and impair seedling emergence. Therefore, achieving a high and uniform rate of seedling emergence after sowing is the first key step toward optimizing yield.

With increasing soil depth, the rate of water evaporation decreases, and the stability of relative soil water content (SWC) increases. As the influence of soil water fluctuation on seed diminishes, there is a corresponding increase in seedling vigor, dry matter accumulation, and allocation to the seedling (Proctor and Sullivan, 2013; Gesch et al., 2017; Ali and Bubaker, 2019). Moderately deep sowing under water-deficit conditions can therefore not only improve seedling emergence vigor but also promote mature plant growth and yield potential (Yagmur and Kaydan, 2009; Cheng et al., 2016; Zuo et al., 2017). However, in the YRB, some years experience a continuous increase in rainfall after rapeseed sowing, resulting in waterlogged soil conditions (National Meteorological Information Center, China Meteorological Administration). This leads to increased stress during seed germination with increased sowing depth (Kanno et al., 2025). In addition, when the sowing depth is too deep, the time required for seed germination and seedling emergence is extended, and the difficulty of seedling emergence increases, along with greater vulnerability to pathogen attack (Zuo et al., 2017; Lamichhane and Soltani, 2020), poor soil aeration, and accumulation of toxic substances, all of which inhibit seed germination and reduce seedling uniformity (Dozet et al., 2020). Moreover, different genotypes exhibit significant differences in tolerance to deep sowing (Sohail et al., 2025). Therefore, determining the optimal sowing depth suitable for different water conditions is a key agronomic factor for crop establishment and yield potential.

Glucosinolates (GS) are a class of secondary metabolites predominantly found in Brassicaceae and play a pivotal role in plant responses to both biotic and abiotic stresses (Jahangir et al., 2009; Xu et al., 2018; Chowdhury, 2022; Luo et al., 2022). Extensive research has demonstrated that GS is a central component of the sophisticated defense system in Brassicaceae (Sharma et al., 2025). Upon hydrolysis by myrosinase, GS yields bioactive compounds such as isothiocyanates and nitriles, which not only exhibit strong antimicrobial and insecticidal activities but also reduce herbivore palatability (Kumar et al., 2010; Sotelo et al., 2015). Beyond their defensive functions against pathogens and herbivores, GS and their hydrolysis products also modulate enzymatic activity, act as antioxidants, and enhance tolerance to abiotic stresses such as drought and salinity through osmotic adjustment and regulation of water transport (Martínez-Ballesta et al., 2015; Gantait et al., 2024). Critically, GS can activate key defense signaling pathways, including those mediated by jasmonic acid (JA) and salicylic acid (SA), thereby serving as integrative hubs that coordinate plant responses to combined biotic and abiotic challenges (Yan and Chen, 2007).

However, in modern rapeseed breeding programs, low seed GS and low erucic acid contents have become standard selection criteria to improve nutritional and feed quality, leading to the predominance of double-low cultivars in cereal-based cropping systems (Beszterda and Nogala-Kałucka, 2019) Unfortunately, this has been accompanied by a gradual decline in seed stress resistance associated with reduced GS content (Ai et al., 2024). Despite their recognized importance, the specific roles of individual GS compounds and their hydrolysis products in mediating responses to abiotic stresses remain poorly understood. Crucially, it is unknown whether exogenous GS application can compensate for reduced endogenous GS in modern cultivars and synergize with optimized sowing depth to improve seedling emergence under moisture-limited or deep-sowing conditions. To our knowledge, this is the first study to integrate optimal sowing depth screening with exogenous GS application in rapeseed. We hypothesized that elevating GS levels could enhance seedling emergence under deep sowing and water stress. Accordingly, we evaluated seedling emergence and early growth across sowing depths (1–5 cm) under four moisture regimes (variable moisture, drought, normal, and waterlogged) and tested whether exogenous GS application mitigates the adverse effects of deep sowing. This study aims to identify agronomically optimal sowing practices and validate GS-mediated biochemical priming as a strategy to improve seedling establishment under challenging field conditions.

## 2 Materials and methods

### 2.1 Materials

This study compared the performance of 16 leading rapeseed cultivars from China, including 13 hybrid and three conventional cultivars (**Supplementary Table 1**).

### 2.2 Experimental design

#### 2.2.1 Optimal sowing depth determination in the field

Sowing depth influences soil moisture availability, seed germination, and early seedling establishment (Proctor and Sullivan, 2013; Gesch et al., 2017; Ali and Bubaker, 2019). However, the optimal sowing depth is modulated by factors such as genotype and soil moisture content (Rebetzke et al., 2007; Kanno et al., 2025). Based on this, we designed the following experiment:

Field trials were conducted during 2019–2020 and 2020–2021 (growing seasons: September to May) at Huanggang Academy of Agricultural Sciences (HG) and Xiangyang Academy of Agricultural Sciences (XY) in Hubei Province. Three rapeseed cultivars, Huayouza62 (H), Zhongshuang11 (Z), and Huayou2101 (Y), were sown under variable moisture conditions (SWC 30%–100%) with three sowing depth treatments (D1: 1 cm, D3: 3 cm, D5: 5 cm).

The same three cultivars (H, Z, Y) were selected for field trials at Huazhong Agricultural University in Wuhan, Hubei Province, during 2021–2022 and 2023–2024. Three water conditions (drought: SWC 30%–60%, normal water conditions: SWC 60%–90%, waterlogged: SWC 90%–100%) were established using rain shelters. The experimental design included the same three sowing depth treatments (D1, D3, and D5) under each water condition.

All treatments had two technical replicates and three biological replicates, each consisting of 50 seeds. The number of seedlings emerging from the soil was recorded after 14 days postsowing (at 21 days under drought stress) to calculate emergence percentage (EP) (**Table 1**), and the morphological characteristics of plants were measured at 2 months after sowing. Nitrogen (N), phosphorus (P), potassium (K), and boron fertilizers were applied before sowing at 9 × 10^−3^ kg/m^2^, 9 × 10^−3^ kg/m^2^, 9 × 10^−3^ kg/m^2^, and 1.5 × 10^−3^ kg/m^2^, respectively. Direct seeding was adopted with a row spacing of 25 cm. Soil physical properties are listed in **Supplementary Table 2**.

**Table 1:** Seedling emergence calculations.

Indices name	Unit	Calculation formula
Emergence percentage (EP)	%	Maximum number of emergent seedlings/total number of seeds * 100%
Emergence index (EI)	–	∑(Et/Dt)
Mean emergence time (MET)	Day	∑(Et * Dt)/∑Et
Root-to-stem ratio (RS)	–	Root length/stem length
Relative emergence percentage (rEP)		EP ratio (3 cm/1 cm) under each water condition
Relative emergence index (rEI)		EI ratio (3 cm/1 cm) under each water condition
Relative mean emergence time (rMET)		MET ratio (3 cm/1 cm) under each water condition

*Et*, number of emergent seeds on day *t*; *Dt*, day after sowing.

#### 2.2.2 Optimal sowing depth determination in pots

To precisely determine the interactive effects of sowing depth and soil water content on seed emergence (Kanno et al., 2025), a greenhouse experiment was conducted using 16 current rapeseed cultivars under three water conditions (drought: SWC 40% ± 5%; normal: SWC 70% ± 5%; waterlogged: SWC 100% ± 5%), with five sowing depth treatments (D1: 1 cm, D2: 2 cm, D3: 3 cm, D4: 4 cm, D5: 5 cm). The design included three biological replicates per treatment, each consisting of 20 seeds. The number of emergent seedlings was counted every day up to 14 days (21 days) after sowing. Seedling EP, emergence index (EI), mean emergence time (MET), relative emergence percentage (rEP), relative emergence index (rEI), and relative mean emergence time (rMET) were calculated. Root length and stem length of seedlings were recorded at 14 days (21 days) to calculate the root-to-stem ratio (RS) (**Table 1**). All greenhouse experiments were conducted in a 25°C/20°C temperature, 75%/70% air humidity, and 13,000/0 lx illumination regime under a 16-h light/8-h dark cycle.

#### 2.2.3 Exogenous application of glucosinolate under different water conditions with moderately deep sowing

At the selected optimal sowing depth (3 cm), we aimed to validate the effects of GS on rapeseed emergence and early growth under varying soil water conditions (Martínez-Ballesta et al., 2015; Gantait et al., 2024). Three conditions were tested—drought (SWC 40% ± 5%), normal (SWC 70% ± 5%), and waterlogged (SWC 100% ± 5%)—with two sowing depths (D1 and D3). Three treatments (ZS11: untreated seed, H_2_O: double-distilled water, GS: 40 mg/L glucosinolates) were applied for seed priming.

The number of emergent seedlings was counted every day up to 14 days (21 days) after sowing to calculate EP (**Table 1**). Morphological characteristics of plants were measured at the eight-leaf stage. All agronomic practices were identical to those described in Section 2.2.1.

##### 2.2.3.1 Seed priming procedure

Zhongshuang11 (ZS11) was used for the greenhouse experiment. Each treatment included three biological repetitions, with 20 seeds per replicate. The seeds were soaked at 25°C and 120 rpm conditions using a temperature shaker (HYG-A, Jiangsu, China) for 10 h and dried at 25°C for about 12 h (Ai et al., 2024).

### 2.3 Soil water content

Soil samples (0–10 cm) were taken from the control plot with no seed, and soil water content was determined using the drying method in an oven for about 1 day at 105°C (Chang et al., 2005).

### 2.4 Plant phenotypic determination

Three plants were selected at the eight-leaf stage for each biological replicate to analyze phenotypic traits. Root and shoot lengths were determined using the cotyledon node as the boundary, root collar diameter was measured with a vernier caliper at the cotyledon node, and total leaf area was measured using a LI-3100C AREA METER (LI-COR Inc., Lincoln, NE, USA). Dry matter was determined by separating plants into shoot and root, followed by oven drying at 105°C for 30 min, then 80°C for 3–5 days until a constant weight was achieved (Bai et al., 2024; Wang et al., 2024).

### 2.5 Data analysis

To evaluate differences among treatments, data for each variable were subjected to analysis of variance using SPSS 25.0 software (SPSS Inc., Chicago, IL, USA). The Levene and Kolmogorov–Smirnov tests were applied to assess the normality and homogeneity of variance. Student’s *t*-test and Duncan’s multiple range test were used to analyze significant differences between treatments. Data from field and pot experiments were analyzed using multivariate analysis of variance. In the field experiment, year, location, and water conditions were treated as random effects to account for uncontrolled environmental variation and to support broader inference. Fixed effects included cultivar and sowing depth. In the pot experiment, only fixed effects (cultivar, sowing depth, and water conditions) were considered due to controlled environmental conditions. Pearson’s correlation coefficient was used for correlation analysis.

To evaluate the variation of 1–5 cm sowing depth under drought conditions, the mean seedling EP of the 16 cultivars was calculated. The regression equation was constructed using Gaussian fitting in SPSS. The regular residual and *F*-test were used to ensure the validity and reliability of the equation.

Data were analyzed using Microsoft Excel 2016 software (Microsoft, Redmond, WA, USA), and graphical representations were generated using Origin 9.0 software (OriginLab Corporation, Northampton, MA, USA).

## 3 Results

### 3.1 Seedling emergence and plant growth under variable moisture conditions at different sowing depths

**Figure 1:**
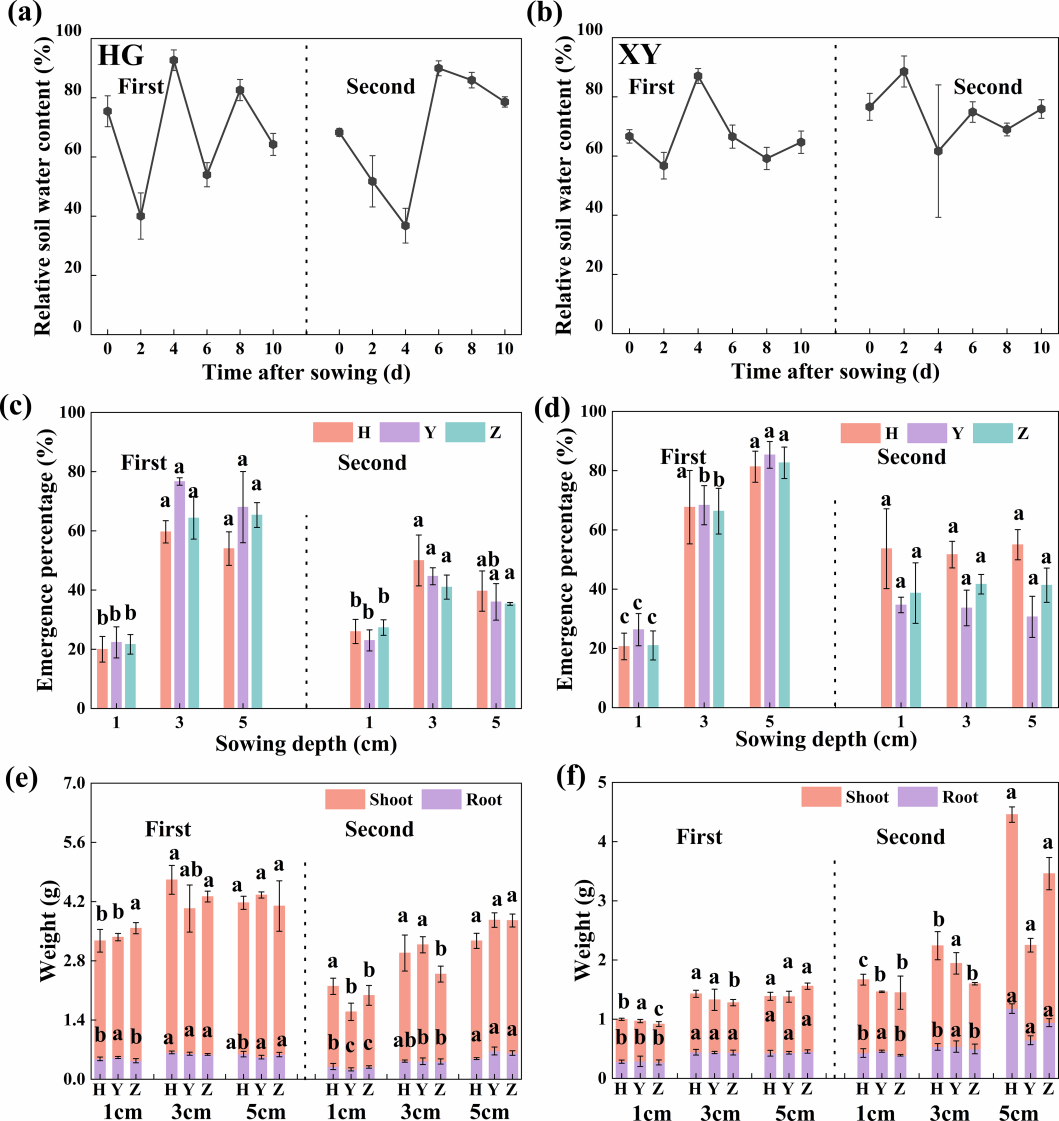
Relative soil water content, seedling emergence, and growth at different sowing depths in Huanggang and Xiangyang. **(a, b)** Relative soil water content of 0–10 cm; **(c, d)** seedling emergence percentage; and **(e, f)** root and shoot dry weight in Huanggang and Xiangyang with three sowing depths (1, 3, and 5 cm), respectively. Bars represent ± SD of replicates. Small letters represent significant differences at the *p* < 0.05 level according to Duncan’s multiple comparisons test. HG, Huanggang; XY, Xiangyang; H, Huayouza62; Y, Huayou2101; Z, Zhongshuang11.

Soil water content is an important factor affecting the seedling emergence under different sowing depths. In this study, seedling emergence and plant growth were investigated at three sowing depths (D1, D3, and D5) under variable moisture conditions in HG and XY. The SWC ranged from 30% to 100% during seedling emergence (**Figures 1a, b**). Furthermore, seedling EP reached a maximum at D3 in Huanggang, while the average EP of the three cultivars over both years increased by 145.8% compared with D1. However, in Xiangyang, seedling EP increased with sowing depth during the first year, and the EP of the three cultivars at D3 was increased by 200.9% than at D1. In contrast, no significant differences (*p* ≥ 0.762) were observed among the three sowing depths during the second year (**Figures 1c, d**).

At the seedling stage, no significant differences (*p* = 0.084) in root length were detected among D1, D3, and D5 in either HG or XY, whereas all other indices were significantly improved (*p* ≤ 0.003) at D3 and D5 compared with D1. For the mean of the three cultivars over both years and trial locations, the average root length increased by 8.6% at D3 compared with D1, as did root collar diameter (19.8%), root dry weight (40.8%), plant height (18.3%), leaf area (21.3%), and shoot dry weight (35.5%) (**Figures 1e, f**; **Supplementary Figure 1**).

### 3.2 Seedling emergence and plant growth under normal water conditions at different sowing depths

Three water conditions—drought, normal, and waterlogged—were established in the field to investigate the effects of sowing depth (D1, D3, and D5) on rapeseed seedling emergence and subsequent plant growth across varying soil moisture levels.

**Figure 2:**
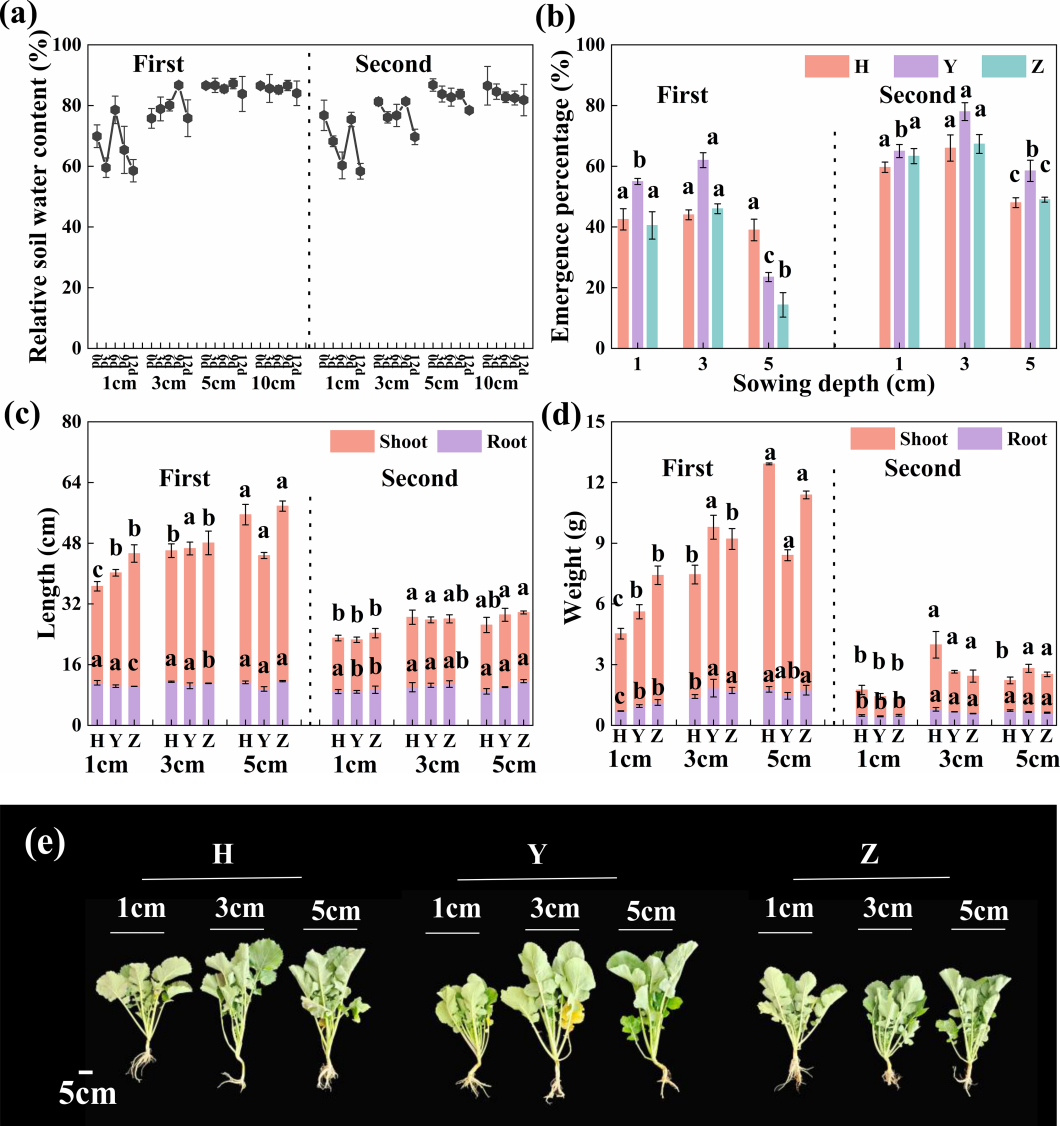
Soil water content, seedling emergence, and growth at different sowing depths under normal water conditions. **(a)** Relative soil water content of 0–10 cm; **(b)** seedling emergence percentage; **(c)** root and shoot length; **(d)** root and shoot dry weight; and **(e)** plant growth at three sowing depths (1, 3, and 5 cm), respectively. Bars represent ± SD of replicates. Small letters represent significant differences at the *p* < 0.05 level according to Duncan’s multiple comparisons test. H, Huayouza62; Y, Huayou2101; Z, Zhongshuang11.

Under normal water conditions in the field experiment, soil water content and its stability increased with soil depth during seedling emergence (**Figure 2a**). Seedling EP increased initially and subsequently decreased as sowing depth increased, reaching a maximum at D3. At this depth, the average EP of the three cultivars increased by 11.1% compared with D1 in both years (**Figure 2b**).

At the seedling stage, no significant differences (*p* ≥ 0.105) were observed in root length or root collar diameter among D1, D3, and D5, while all other indices were significantly greater (*p* ≤ 0.012) at D3 and D5 compared with D1. Furthermore, root length increased by 9.9% across the three cultivars over both years at D3 compared with D1, as did root collar diameter (22.1%), root dry weight (62.7%), plant height (22.4%), leaf area (53.8%), and shoot dry weight (86.1%) (**Figures 2c–e**; **Supplementary Figure 2a, b**).

### 3.3 Seedling emergence and plant growth under drought stress at different sowing depths

**Figure 3:**
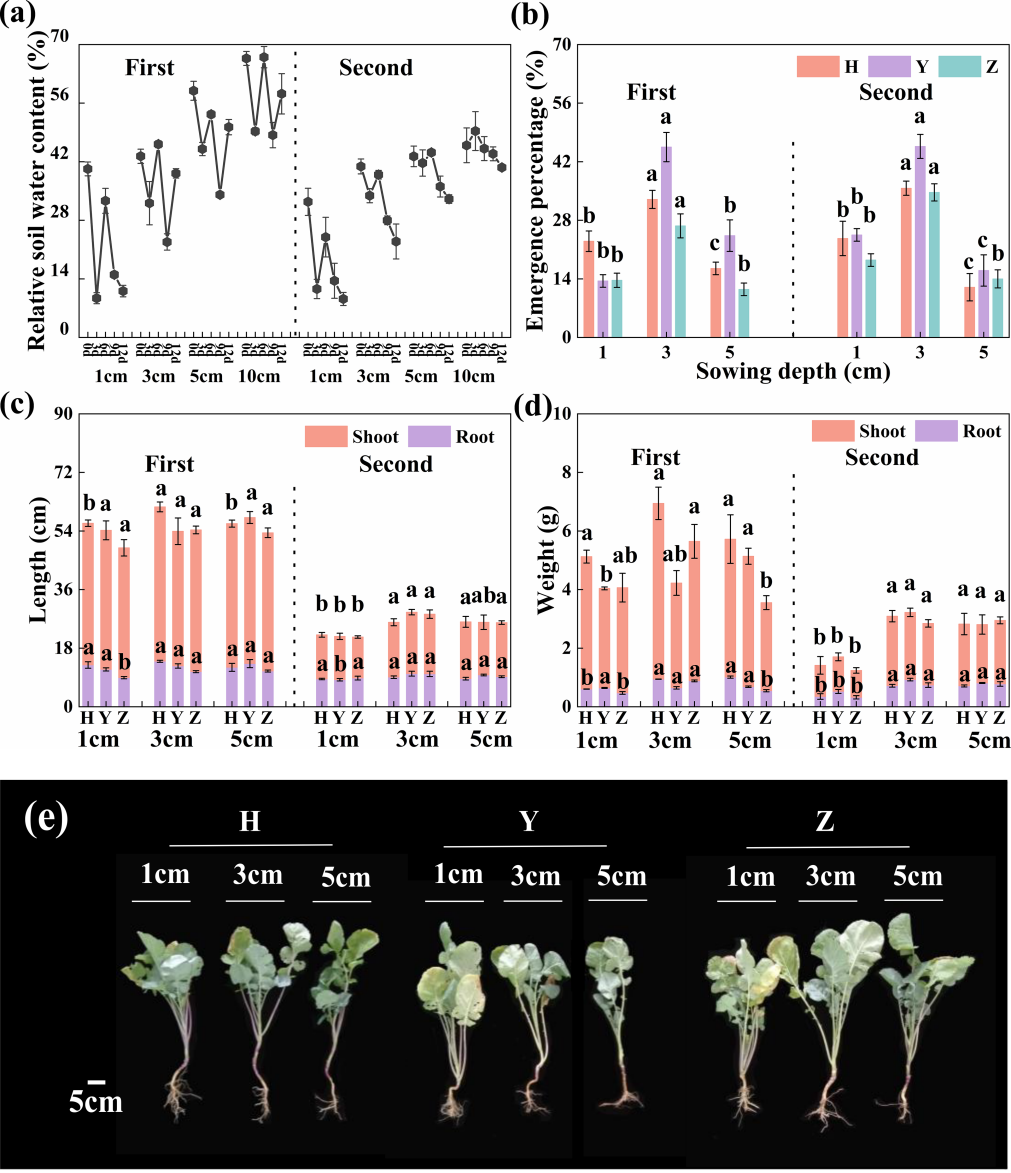
Soil water content, seedling emergence, and growth at different sowing depths under drought stress. **(a)** Relative soil water content of 0–10 cm; **(b)** seedling emergence percentage; **(c)** root and shoot length; **(d)** root and shoot dry weight; and **(e)** plant growth at three sowing depths (1, 3, and 5 cm), respectively. Bars represent ± SD of replicates. Small letters represent significant differences at the *p* < 0.05 level according to Duncan’s multiple comparisons test. H, Huayouza62; Y, Huayou2101; Z, Zhongshuang11.

Under drought stress in the field experiment, soil water content and its stability increased with soil depth, with the most pronounced fluctuations occurring at 1 cm during seedling emergence (**Figure 3a**). As sowing depth increased, seedling EP exhibited an initial increase followed by a decline, with the highest value observed at D3. The average EP of the three cultivars increased by 100% at D3 compared with D1 in both trial years (**Figure 3b**).

At the seedling stage, there were no significant differences (*p* ≥ 0.082) in root length and shoot dry weight among D1, D3, and D5, while all other indices were significantly greater (*p* ≤ 0.024) at D3 and D5 compared with D1. Moreover, for the mean of the three cultivars over both years, the average root length increased by 13.7% at D3 compared with D1, as did root collar diameter (18.2%), root dry weight (75.9%), plant height (21.1%), leaf area (63.9%), and shoot dry weight (69.6%) (**Figures 3c–e**; **Supplementary Figure 2c, d**).

### 3.4 Seedling emergence and plant growth under waterlogged stress at different sowing depths

**Figure 4:**
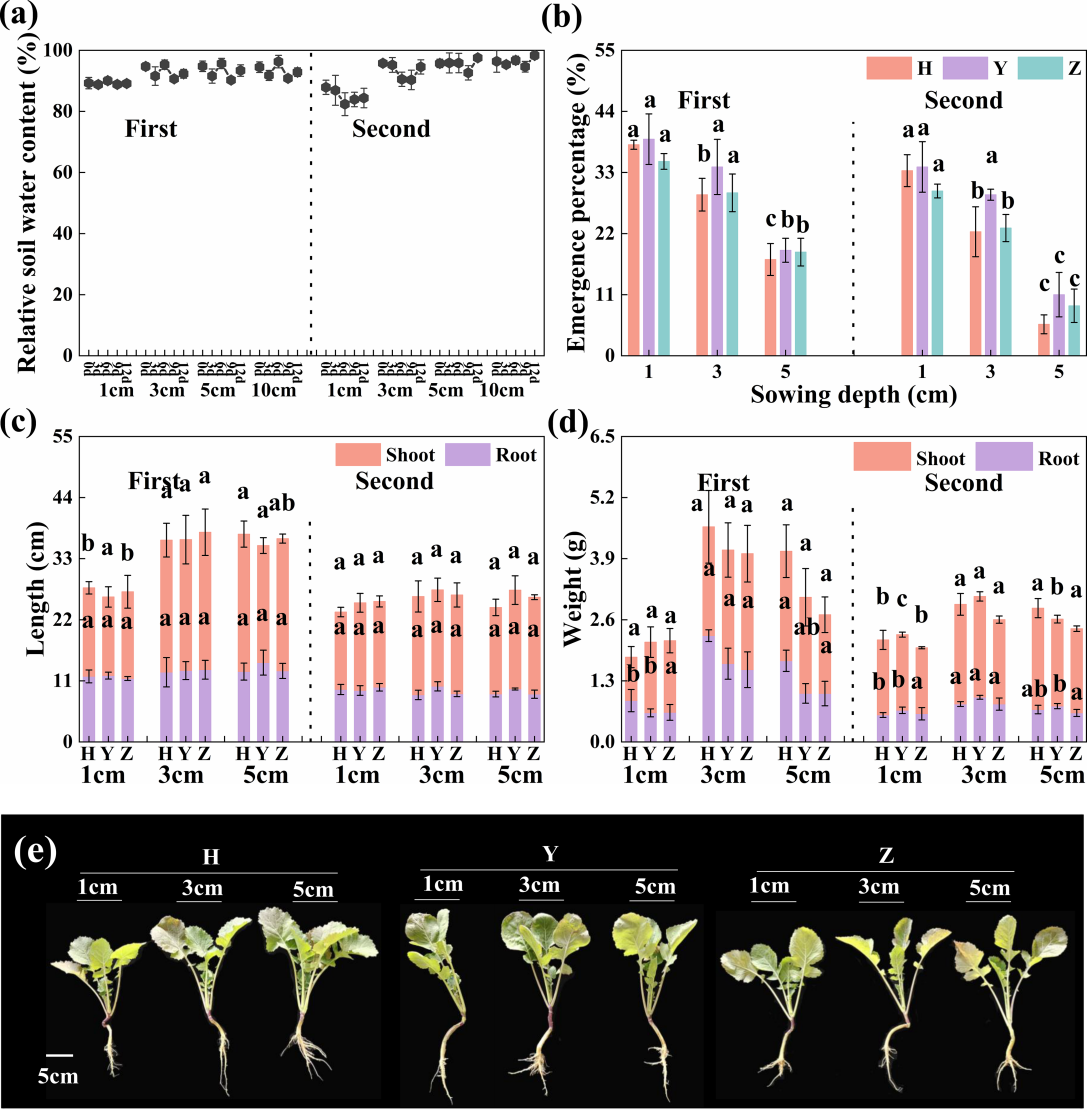
Soil water content, seedling emergence, and growth at different sowing depths under waterlogged stress. **(a)** Relative soil water content of 0–10 cm; **(b)** seedling emergence percentage; **(c)** root and shoot length; **(d)** root and shoot dry weight; and **(e)** plant growth at three sowing depths (1, 3, and 5 cm), respectively. Bars represent ± SD of replicates. Small letters represent significant differences at the *p* < 0.05 level according to Duncan’s multiple comparisons. H, Huayouza62; Y, Huayou2101; Z, Zhongshuang11.

Under waterlogged stress in the field experiment, soil water content exhibited little variation across soil layers during seedling emergence (**Figure 4a**). Overall, seedling EP decreased as sowing depth increased, although there were no significant differences (*p* ≥ 0.095) for cultivars HY2101 and ZS11 during the first year, or for HY2101 during the second year, at D3 compared with D1. However, the EP of all three cultivars was significantly lower (*p* < 0.001) at D5 than at D1 (**Figure 4b**).

At the seedling stage, no significant differences (*p* ≥ 0.056) were observed in root length and leaf area among D1, D3, and D5, while all other indices were significantly increased (*p* ≤ 0.018) at D3 and D5 compared with D1. Meanwhile, for the mean of the three cultivars over both years, average root length increased by 2.2% for D3 compared with D1, as did root collar diameter (32.3%), root dry weight (97.9%), plant height (37.7%), leaf area (30.4%), and shoot dry weight (61.2%) (**Figures 4c–e**; **Supplementary Figure 2e, f**).

### 3.5 Soil water and sowing depth affect seedling emergence and vigor

In the pot experiment, 16 rapeseed cultivars were grown under three water conditions (drought, normal, and waterlogged) at five sowing depths (D1, D2, D3, D4, and D5). Variation in their responses was evaluated using curve-fitting analysis to determine the optimal sowing depth under drought stress.

**Figure 5:**
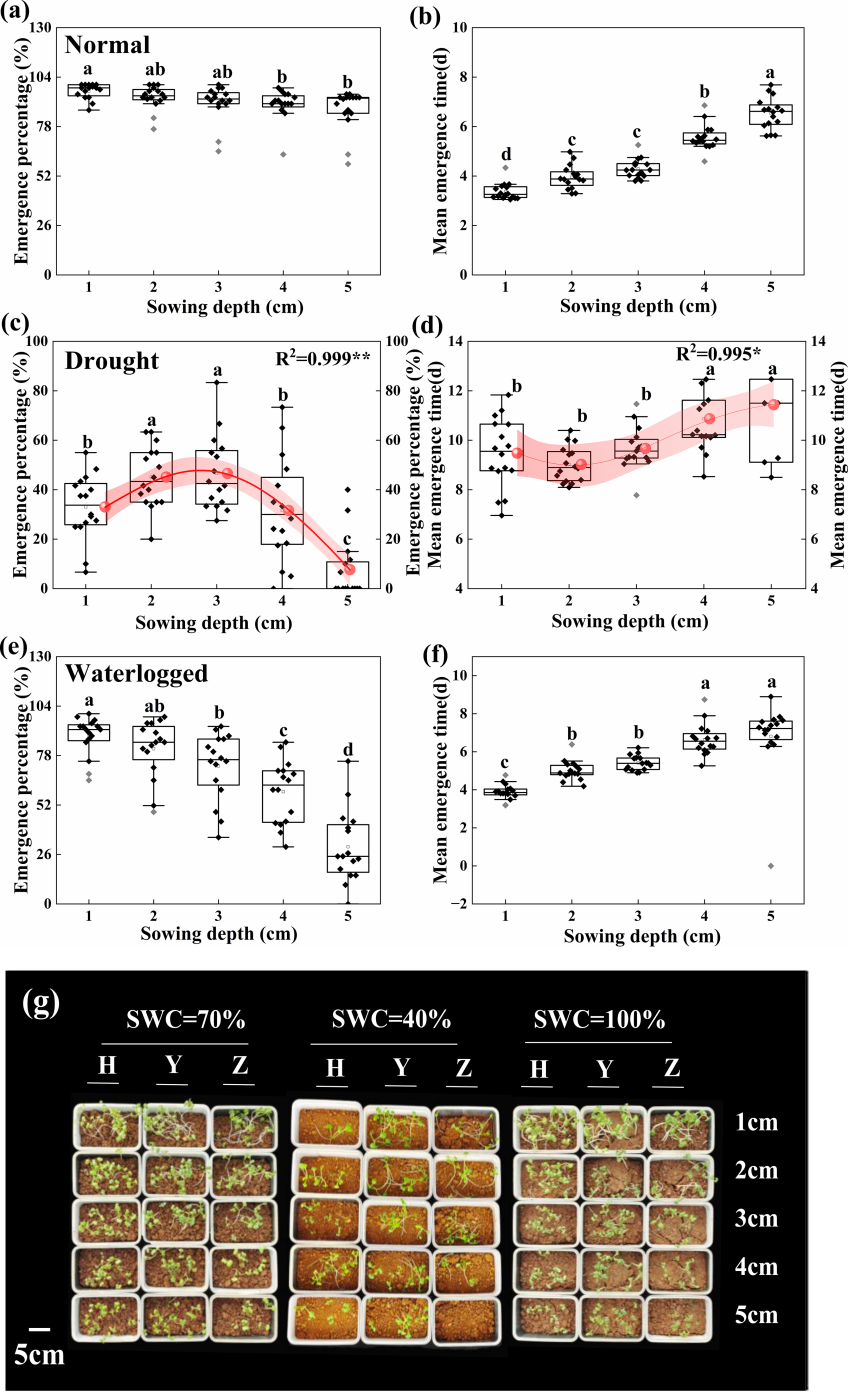
Seedling emergence ability at different sowing depths under different water contents. Seedling emergence percentage and mean emergence time at five sowing depths (1, 2, 3, 4, and 5 cm) under **(a, b)** drought (SWC = 40% ± 5%), **(c, d)** normal water (SWC = 70% ± 5%), and **(e, f)** waterlogged (SWC = 100% ± 5%) conditions, respectively. **(g)** Seedling emergence and growth under three water conditions. Seedling EP and ET of all cultivars at five sowing depths were evaluated by curve-fitting analysis under drought conditions. *R*^2^ represents the correlation of the equation, and asterisks indicate significant differences (^*^p < 0.05; ^**^p < 0.01). Small letters represent significant differences at the *p* < 0.05 level according to Duncan’s multiple comparisons test. H, Huayouza62; Y, Huayou2101; Z, Zhongshuang11.

Under normal water conditions, seedling emergence began from 2 to 4 days after sowing, with EP reaching a maximum at 8–10 days across all five depths (**Supplementary Figure 3a**). As sowing depth increased, EP decreased slightly, but no significant differences (*p* ≥ 0.229) were detected among D1, D2, and D3 in the majority of cultivars. Specifically, 15 cultivars exhibited EP > 90% at D1, as dis 14 at D2, 13 at D3, 12 at D4, and 10 at D5 (**Figure 5a**). Compared with D1, the average EI across all cultivars decreased by 24.9%, and seedling RS decreased, while MET increased by 0.9 day at D3 (**Figures 5b, g**; **Supplementary Figure 3b, c**).

Under drought stress, emergence began from 5 to 7 days after sowing for the five depths, with seedling EP reaching a maximum at approximately 15 days for D1 to D4 and 20 days for D5 (**Supplementary Figure 3d**). As sowing depth increased, both seedling EP and EI initially increased and subsequently decreased, with a maximum at D3 and D2, while the lowest MET was at D2, with no significant differences (*p* = 0.207) among D1, D2, and D3. Nonlinear fitting indicated that EP, EI, and MET would reach maximal values at sowing depths of 2.5, 2.5, and 2 cm. However, EP and EI at D2 and D3 were significantly higher (*p* ≤ 0.020) than at D1, with EP increasing by 36.6% at D2 and 41% at D3 (**Figures 5c, d**; **Supplementary Figure 3e**). Seedling RS also decreased with increasing sowing depth (**Figure 5g**; **Supplementary Figure 3f**).

Under waterlogged stress, emergence began 3 to 5 days after sowing, with EP reaching a maximum at 9–11 days across all five depths (**Supplementary Figure 3g**). As sowing depth increased, seedling EP decreased, with a sharp drop observed at D4 and D5. Significant differences in EP were detected among cultivars at D2–D5, whereas no significant differences were found between D1 and D3 among eight cultivars (**Figure 5e**). Moreover, compared with D1, the average EI decreased by 43.0% and MET was delayed by 1.5 days at D3, accompanied by a reduction in seedling RS (**Figures 5f, g**; **Supplementary Figure 3h, i**).

Overall, a relatively high level of soil water stability at D3 was conducive to seedling emergence under drought and normal water conditions. Under waterlogged stress, EP at D3 was not significantly different for half of the cultivars. However, excessively deep sowing (D5) was detrimental to seedling emergence under all conditions. Although plant growth parameters generally improved with increasing sowing depth (D1 to D5), the highest combined performance in seedling emergence and early growth was observed at D3 under drought and normal water conditions.

### 3.6 Interactive effects of multiple factors and seed composition on tolerance to deep sowing

In the field experiment, location, year, SWC, sowing depth, and cultivar all significantly influenced seedling emergence and early growth. Specifically, both year and sowing depth had significant effects on all measured indices. Moreover, the interaction effects of year × SWC and year × sowing depth were also significant for all indices. In the pot experiment, SWC, sowing depth, and cultivar significantly affected seedling EP, EI, and MET. Furthermore, the two-way interactions (SWC × sowing depth, SWC × cultivar, sowing depth × cultivar), as well as the three-way interaction (SWC × sowing depth × cultivar), were statistically significant for these three indices (**Table 2**).

**Table 2:** Multivariate analysis of variance.

Experiment type	Factors	EP	EI	MET	Shoot length	Root length	Root collar diameter	Leaf area	Shoot weight	Root weight
Field	L	**	–	–	**	**	**	**	**	NS
	Y	**	–	–	**	**	**	**	**	**
	WC	**	–	–	**	NS	**	**	**	**
	SD	**	–	–	**	**	**	**	**	**
	C	**	–	–	NS	NS	**	NS	**	**
	L×Y	NS	–	–	**	**	**	**	**	**
	L×SD	**	–	–	NS	NS	NS	NS	*	NS
	L×C	**	–	–	NS	NS	NS	NS	NS	NS
	Y×WC	**	–	–	**	**	**	**	**	**
	Y×SW	**	–	–	**	*	**	**	**	**
	Y×C	**	–	–	NS	**	**	**	**	**
	WC×SD	**	–	–	**	NS	**	**	**	**
	WC×C	NS	–	–	**	**	**	**	**	**
	SD×C	NS	–	–	NS	NS	NS	*	**	*
	L×Y×SD	**	–	–	*	NS	NS	NS	NS	NS
	L×Y×C	NS	–	–	NS	NS	NS	NS	NS	NS
	L×SD×C	NS	–	–	NS	NS	NS	NS	NS	NS
	Y×WC×SD	**	–	–	**	NS	**	**	**	**
	Y×WC×C	NS	–	–	**	*	**	**	**	**
	Y×SD×C	NS	–	–	NS	NS	NS	**	**	NS
	WC×SD×C	*	–	–	NS	NS	NS	NS	**	NS
	L×Y×SD×C	NS	–	–	NS	NS	NS	NS	NS	NS
	Y×WC×SD×C	NS	–	–	**	NS	NS	**	**	NS
Pot	WC	**	**	**	–	–	–	–	–	–
	SD	**	**	**	–	–	–	–	–	–
	C	**	**	**	–	–	–	–	–	–
	WC×SD	**	**	**	–	–	–	–	–	–
	WC×C	**	**	**	–	–	–	–	–	–
	SD×C	**	**	**	–	–	–	–	–	–
	WC×SD×C	**	**	**	–	–	–	–	–	–

L, location; Y, year; W, relative soil water content; S, sowing depth; C, cultivar. Asterisks indicate significant differences (NSP>0.05, *P < 0.05, **P < 0.01).

To further investigate the role of cultivar in seedling emergence under deep sowing, the relationships between seed composition of 16 rapeseed cultivars and rEP, rEI, and rMET were analyzed under drought, normal, and waterlogged conditions in the pot experiment. The results showed that, under drought stress, both seedling rEP and rMEI were significantly and positively correlated with palmitic acid and linolenic acid contents. Under waterlogged conditions, seedling rMET was significantly and positively correlated with palmitic acid content (**Table 3**).

**Table 3:** Correlation analysis between seed composition and emergence percentage under different water conditions with moderately deep sowing.

Water Conditions	Indices	Protein	Fat	Palmitic Acid	Oleic acid	Linoleic acid	Linolenic acid	Glucosinolate	Erucic acid
Drought	rEP	0.096	-0.057	0.573*	-0.368	0.383	0.588*	-0.168	-0.268
	rEI	0.023	0.009	0.509*	-0.377	0.387	0.558*	-0.17	-0.18
	rMET	0.231	-0.11	0.143	-0.09	0.197	0.36	-0.137	-0.398
Normal	rEP	0.473	-0.373	0.416	-0.241	0.204	0.219	0.123	-0.238
	rEI	0.464	-0.439	0.382	-0.114	0.086	0.196	0.108	-0.299
	rMET	-0.276	0.263	-0.018	-0.304	0.268	0.042	-0.023	0.282
Waterlogged	rEP	0.16	-0.185	-0.025	-0.009	-0.044	-0.057	0.173	-0.054
	rEI	-0.101	0.175	-0.275	0.008	0.021	0.006	0.006	-0.052
	rMET	0.422	-0.474	0.637**	-0.131	0.029	0.185	0.25	-0.192

rEP, relative emergence percentage; rEI, relative emergence index; rMET, relative mean emergence time. Asterisks indicate significant differences (^*^P < 0.05, ^**^P < 0.01).

### 3.7 Exogenous application of glucosinolate improves seedling emergence and plant growth under different water conditions with moderately deep sowing

**Figure 6:**
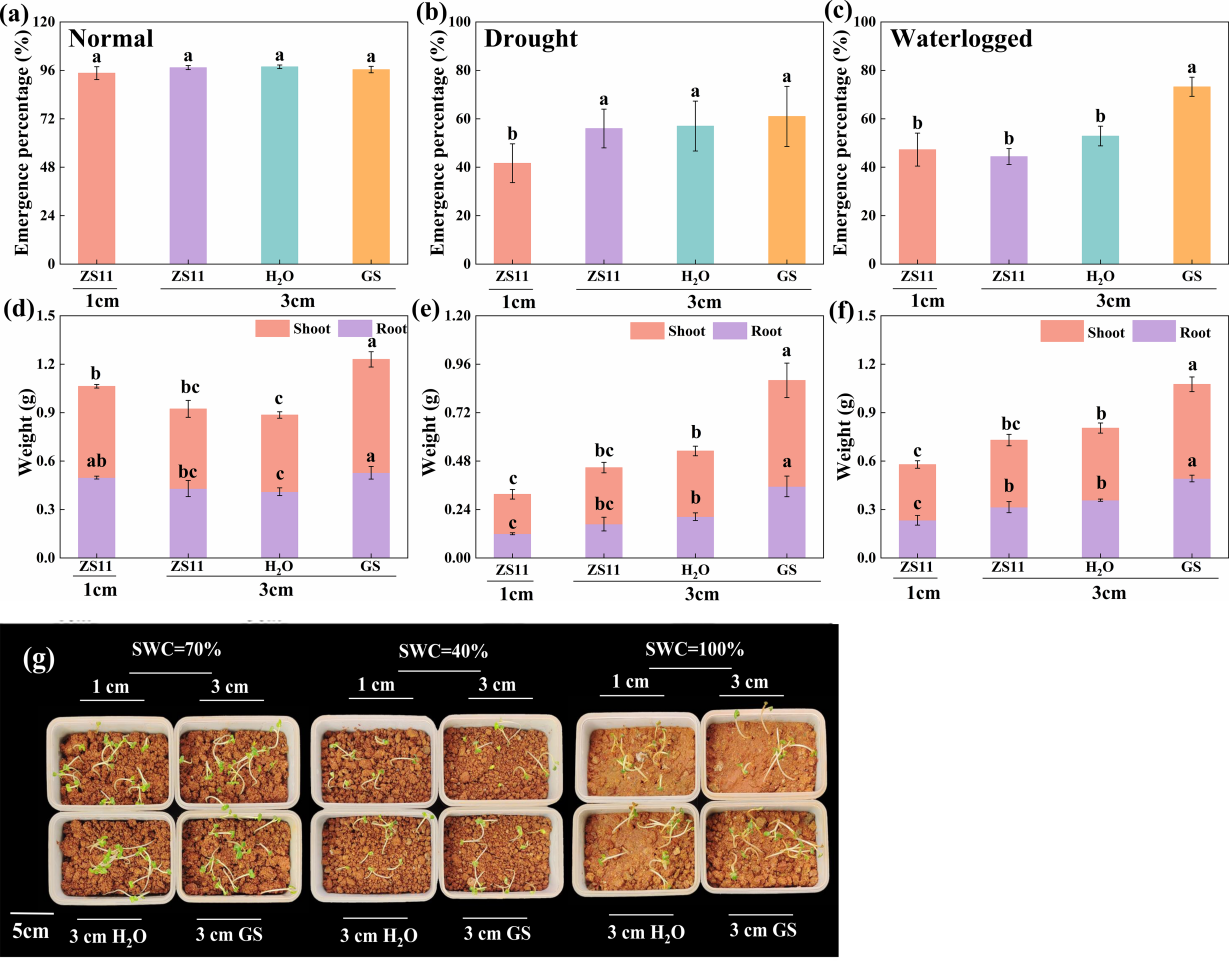
Exogenous application of GS improves seedling emergence and plant growth under different water conditions with moderately deep sowing. Seedling emergence percentage and shoot and root dry weight under **(a, d)** normal water, **(b, e)** drought, and **(c, f)** waterlogged conditions at 1 and 3 cm sowing depths after seed priming with GS, respectively. **(g)** Seedling emergence under three water conditions after seed priming with GS. Bars represent ± SD of replicates. Small letters represent significant differences at the *p* < 0.05 level according to Duncan’s multiple comparisons test. ZS11, untreated seed; H_2_O, double-distilled water; GS, glucosinolate.

To validate our hypothesis, we evaluated seedling emergence and plant growth at the optimum sowing depth (D3) under drought, normal water, and waterlogged conditions following seed priming with GS. Under normal water conditions, no significant differences (*p* = 0.524) were observed between H_2_O and GS treatments (**Figures 6a, g**). In contrast, under drought and waterlogged stress, seedling EP at D3 with GS treatment significantly increased (*p* ≤ 0.048) compared with D1, by 46.4% and 63.0%, respectively (**Figures 6b, c, g**).

Regarding plant growth at D3 with GS treatment versus D1, under normal water conditions, there were no significant differences in root length, root dry weight, and root collar diameter (*p* ≥ 0.050), whereas all other indices were significantly greater (*p* ≤ 0.036), with increases of 15.1% (plant height), 30.1% (leaf area), and 24.2% (shoot dry weight) (**Figure 6d**; **Supplementary Figure 4a–c**). Under drought stress, all indices were significantly enhanced (*p* ≤ 0.022), showing increases of 12.2% (root length), 43.8% (root collar diameter), 195.4% (root dry weight), 24.8% (plant height), 172.6% (leaf area), and 168.8% (shoot dry weight) (**Figures 6e**, **Supplementary Figure 4d–f**). Under waterlogged stress, root length showed no significant differences (*p* = 0.167), but all other indices were significantly increased (*p* ≤ 0.003), with improvements of 29.5% (root collar diameter), 111.0% (root dry weight), 24.3% (plant height), 85.1% (leaf area), and 68.8% (shoot dry weight) (**Figure 6f**; **Supplementary Figure 4g–i**).

## 4 Discussion

### 4.1 Moderately deep sowing is a key determinant for improving the stability of seedling emergence under different water conditions

Water availability is a critical determinant of seed vigor. In our study, compared with normal water availability, both drought and waterlogged conditions reduced seedling EP and EI while prolonging MET, a pattern consistent with earlier findings (Llanes et al., 2016; Queiroz et al., 2019; Shen et al., 2020). Our results corroborate this response: as soil water content increases, seedling emergence initially rises but then declines sharply under waterlogged conditions (Khaeim et al., 2022).

Previous studies have demonstrated that surface soil moisture exhibits substantial variability, and deep sowing under drought conditions is an effective strategy to promote seed germination and seedling establishment (Cheng et al., 2025). Our results show that, in the pot experiment, as sowing depth increased, there was an initial increase in seedling EP and EI, followed by a decrease, with MET showing an inverse pattern. At 2.5 cm, both EP and EI reached a maximum, while MET reached a minimum. Meanwhile, in the field experiment, seedling EP was significantly higher at D3 than at D1, whereas no significant differences were observed between D1 and D5, indicating that excessively deep sowing is also detrimental for seedling emergence (Cheng et al., 2025; Kanno et al., 2025). The decline in O_2_ availability with increasing soil depth likely contributes to insufficient activation of energy reserves during seed germination and early seedling growth (Conlin and Driessche, 2000), thereby increasing the risk of seedling mortality once available energy sources are depleted (Li et al., 2020). Similarly, under OP conditions, seedling EP in the field initially increased and subsequently declined. Consequently, even when the SWC in the 1–10-cm layer was at 60%–90%, it may still be insufficient to meet the moisture requirement for germination of shallow-sown seeds (Padilla and Pugnaire, 2007; Pinto et al., 2016).

Deep sowing is detrimental to seedling emergence and growth under waterlogged conditions (Kanno et al., 2025). In our study, both EP and EI decreased, while ET increased with increasing sowing depth. Nevertheless, considerable variation existed among cultivars: for half of the 16 cultivars, seedling EP showed no significant difference between D3 and D1. These findings were validated in the field experiment, although the magnitude of EP reduction with sowing depth under waterlogged stress was less pronounced than in the pot experiment. This may be attributed to loose soil structure and enhanced aeration, as well as greater ventilation resulting from straw returning, which alleviates the adverse effects of soil mechanical resistance on seedling emergence (Özmerzi et al., 2002; Zheng et al., 2019). This observation aligns with the notion that genetic background plays a critical role in determining seedling tolerance under stress conditions (Pan et al., 2023; Sohail et al., 2025).

Furthermore, our correlation analysis shows that seed palmitic and linolenic acid contents positively influence emergence from deeper sowing under drought, linking fatty acid composition to early stress resilience (Bai et al., 2024). Plants often increase fatty acid unsaturation under abiotic stress, particularly linolenic acid (He and Ding, 2020). α-Linolenic acid enhances germination and seedling growth under drought by modulating α-amylase activity and remodeling the mobility of the cell membrane (Li et al., 2018). Palmitic acid contributes to phospholipid structure and membrane polarity but appears to have limited direct roles in stress tolerance (Zhao and Qin, 2005; Zhukov, 2015). Notably, some cultivars with high linolenic acid showed no improvement in emergence, indicating that fatty acid composition alone is insufficient to confer stress tolerance. This variability likely reflects differences in other traits, such as seed size, antioxidant capacity, or reserve mobilization efficiency (Pan et al., 2021; Wiraguna, 2022), which interact with fatty acid profiles to determine overall resilience. From a breeding standpoint, selecting for high linolenic acid may benefit only certain genetic backgrounds. Linolenic acid can therefore serve as a valuable indicator for improving deep-sowing tolerance under water-limited conditions, but it should be integrated with other complementary traits in a holistic selection strategy.

In summary, a sowing depth of 3 cm is generally recommended as optimal for achieving robust seedling vigor in rapeseed under different water conditions. Although some studies have suggested that 1 cm is an optimum sowing depth, this recommendation primarily applies to transplanted systems and is unsuitable for direct-seeded rapeseed, as shallow sowing renders seeds vulnerable to rapid fluctuations in surface soil moisture, particularly under drought conditions (Harker et al., 2012; Gesch et al., 2017). These findings inform agronomic practices for optimizing seedling emergence across diverse soil water regimes.

### 4.2 Moderately deep sowing promotes rapeseed plant growth

The interaction between early growth status and environmental conditions is of significant importance for simulating crop growth and yield under extreme climatic scenarios (Bao et al., 2025). A well-developed root system is beneficial to plant growth, enhancing the ability to absorb water and nutrients (Asch et al., 2005; Hodge et al., 2009) and reducing lodging risk (Zuo et al., 2017). Under deep sowing conditions, seedling emergence is driven by rapid hypocotyl elongation but is accompanied by slower root growth, resulting in a significantly reduced early seedling RS. Although seedlings may attempt to promote root growth in response to adverse water stress, root development is often inhibited in deeply sown environments, leading to weak early seedling growth (Grossnickle, 2005).

Interestingly, rapeseed plant growth was promoted at the eight-leaf stage with increasing sowing depth. Shoot length, root collar diameter, leaf area, and shoot and root dry weight were all greater at D3 than at D1. This finding aligns with previous studies showing that rapeseed growth initially increased and subsequently decreased as sowing depth increased, with moderately deep sowing improving plant growth and vigor in arid regions (Yagmur and Kaydan, 2009; Cheng et al., 2016). Although seedling EP under deep sowing is slightly delayed compared with shallow sowing, leading to reduced seedling growth and dry matter accumulation during the stem elongation stage, the onset of self-shading also appears to be delayed after emergence. This delay may alleviate intraspecific competition among rapeseed plants, thereby compensating for deficiencies in early seedling growth (Zimmermann et al., 2010). The compensation effect, however, cannot offset the damage caused by excessively deep sowing (Aikins and Afuakwa, 2008). In our study, little difference in root length was observed between D1 and D2, possibly due to irreversible damage to the root tip induced by deep sowing (Koyama et al., 2001). Nevertheless, root systems established under deeper sowing conditions are better positioned to access water and nutrients from deeper soil layers, which can ultimately promote root growth and development (Cheng et al., 2020). Indeed, both root collar diameter and root dry weight were significantly higher at D3 than at D1, indicating that deep sowing may stimulate radial root expansion and lateral root formation (Cao et al., 2015). These findings suggest that although deep sowing temporarily suppresses early seedling root development, it is beneficial for subsequent plant growth.

### 4.3 Exogenous application of GS improves stress tolerance during seedling emergence and plant growth

High-GS accessions or seed priming with GS improve seed germination under drought conditions (Ai et al., 2024). Similarly, following GS treatment, seedling EP significantly increased at D3 under drought and waterlogged conditions. In addition, exogenous application of GS promoted the plant growth under drought, normal, and waterlogged conditions when combined with deep sowing.

Research has shown that plants under drought and waterlogged stress accumulate higher levels of GS, which may contribute to improving cellular osmoregulation (Schreiner et al., 2009; Brazel et al., 2021). Furthermore, abiotic stress induces the relocalization of GS from the vacuole to the cytosol, thereby increasing its availability for hydrolysis by myrosinases. The hydrolysis products of GS evoke ion channel inhibition and stomatal closure, preventing water loss (Zhao et al., 2008). GS has also been reported to increase the activity of antioxidant enzymes and enhance antioxidant capacity (Gantait et al., 2024). Moreover, exogenous GS affects biomass accumulation during plant growth and appears to be associated with sucrose accumulation (Francisco et al., 2016). GS and their degradation products not only act directly on the plant but also actively modulate the rhizosphere microbial environment (Siebers et al., 2018), similar to the effects of nitrate and schwertmannite (Chen et al., 2025).

However, it should be noted that although exogenous GS application has demonstrated positive effects on seed germination and plant growth, the underlying physiological and molecular mechanisms remain insufficiently substantiated by direct experimental evidence. Consequently, the explanations regarding GS-mediated modulation of osmoregulation, hormone metabolism, antioxidant systems, or rhizosphere microbial communities should be considered putative mechanisms that warrant further investigation. At this stage, a more cautious interpretation is that GS treatment is associated with enhanced stress tolerance and improved growth, while the precise modes of action require deeper mechanistic exploration.

### 4.4 Modulation of sowing depth in rapeseed production

Accurate control of sowing depth is a key approach to improving seedling vigor and crop establishment (Masilamani et al., 2023). It is generally believed that small-seeded species with limited energy reserves, such as rapeseed, are suitable for shallow sowing (1 cm), although this may often result in irregular seedling emergence, large sowing losses, and higher production costs (Håkansson et al., 2013). Our study provides the first evidence that moderately deep sowing (3 cm) can ensure optimal seedling emergence under both normal and drought conditions. However, under waterlogged stress, differences in seedling emergence among varieties were markedly amplified at greater sowing depths. Hosseini et al. (2009) similarly reported that seedling emergence, the time when seed began to emerge, and early seedling growth under different soil water conditions were all affected by seed genotype (Hosseini et al., 2009). Therefore, screening rapeseed cultivars for their ability to maintain high EP under moderately deep sowing combined with waterlogged stress could enhance seed tolerance to water stress (Xu et al., 2011; Nabloussi et al., 2019; Ahmad et al., 2022). In addition, our study demonstrated that seed priming with GS further improves seedling emergence and growth under different water conditions with moderately deep sowing (3 cm), especially under waterlogged conditions. Compared with 1 cm, although the cost increased by 5.5%, the seedling EP increased by 46.4% and 63.0% under drought and waterlogged conditions, respectively. Therefore, under the same density conditions, the total cost decreased by 27.9% and 35.3%. These findings suggest that integrating moderately deep sowing with GS seed priming is likely to further enhance seedling vigor across diverse water conditions while reducing overall production costs.

Mechanization, along with precision and intelligent production methods, is increasingly being adopted in agricultural development. Manual dibbers have been widely used in field production to control seed depth. Traditional dibber drills, however, are often time-consuming, labor-intensive, and aggravate soil compaction, which increases the difficulty of seedling emergence; they are thus unsuitable for small-seeded crops such as rapeseed (Bagautdinov et al., 2018; Agrawal, 2021). With the development of mechanized production, current precision cone drills have been optimized and widely adopted for many crops, with sowing depth and spacing more accurately controlled by pressure-sensitive sensors (Özmerzi et al., 2002; Romaneckas et al., 2022). In addition, the application of unmanned aerial vehicle (UAV) sowing technology minimizes soil disturbance caused by mechanized drills and can reduce sowing cost and time due to lower energy consumption and increased sowing speed (Yamunathangam et al., 2020; Qi et al., 2022). However, sowing depth control remains poorly regulated with current UAV systems. We therefore recommend the widespread adoption of moderately deep sowing for the mechanized production of rapeseed using precision cone drills.

Soil physical properties greatly affect sowing quality, especially soil water content. Under conditions of excess water, machinery operation becomes difficult, and sowing depth uniformity decreases. When water is deficient, seeds struggle to imbibe sufficient moisture. It is therefore necessary to irrigate or rely on rainfall after sowing; however, this can induce soil surface crusting and compaction, further impeding seed emergence (Obour et al., 2019). We suggest maintaining relative soil water content at no less than 40% when using cone drills to achieve moderate sowing depth, ensuring both machinery operability and successful seedling emergence. In addition, soil clod size should be moderately small, as large clods can adversely affect sowing depth, seedling emergence, and plant growth (Bai et al., 2024).

## 5 Conclusion

Moderately deep sowing of rapeseed is an important strategy for improving water adaptability during seedling emergence and early growth. In the pot experiment, results showed that seedling EP reached a maximum at 3 cm sowing depth under both drought and normal conditions. For many cultivars, no significant differences in seedling EP were observed between 1 and 3 cm under waterlogged stress, although subsequent plant growth was more vigorous at 3 cm. Notably, when seeds were primed with glucosinolate at a 3-cm sowing depth, both seedling EP and plant growth were improved compared with 1 cm under drought and waterlogged conditions. In conclusion, a sowing depth of 3 cm with exogenous application of glucosinolate is beneficial for seedling vigor and crop establishment for direct-sown rapeseed.

## Data Availability

The original contributions presented in the study are included in the article/**Supplementary Material**. Further inquiries can be directed to the corresponding author.
