# Correction: Mitigation of soil water stress by moderately deep sowing and exogenous application of glucosinolates during the early seedling stage in rapeseed

**DOI:** 10.3389/fpls.2026.1837656

**Published:** 2026-04-09

**Authors:** Chenyang Bai, Yizhong Lei, Maria Batool, Ali Mahmoud El-Badri, Ying Chang, Jie Kuai, Bo Wang, Jie Zhao, Zhenghua Xu, Sumera Anwar, Graham John King, Jing Wang, Guangsheng Zhou

**Affiliations:** 1 Ministry of Agriculture (MOA) Key Laboratory of Crop Ecophysiology and Farming System in the Middle Reaches of the Yangtze River, College of Plant Science & Technology, Huazhong Agricultural University, Wuhan, China; 2 Field Crops Research Institute, Agricultural Research Center (ARC), Giza, Egypt; 3 Department of Botany, Government College Women University, Faisalabad, Pakistan; 4 Southern Cross Plant Science, Southern Cross University, Lismore, NSW, Australia

**Keywords:** abiotic stress, canola, crop establishment, seedling vigor, sowing depth

An incorrect grant number was provided for “2024YED2300300”. The correct number is “2024YFD2300300”.

The original version of this article has been updated.

