# Supplementary material for: Mitigation of soil water stress by moderately deep sowing and exogenous application of glucosinolates during the early seedling stage in rapeseed
Source: Front Plant Sci. 2026 Mar 6;17:1752750. doi: 10.3389/fpls.2026.1752750 (PMC13062686; doi:10.3389/fpls.2026.1752750)
Supplement: Supplementary file 1 [file DataSheet1.docx]

Table S1 Detailed information of the sixteen rapeseed cultivars

| Cultivar name | Abbreviation | Ecotype | Region |
| --- | --- | --- | --- |
| Huayouza62 | H | semi-winter | Hubei, China |
| Zhuangshuang11 | Z | semi-winter | Hubei, China |
| Huayou2101 | Y | semi-winter | Hubei, China |
| Xiangzayou518 | XZY518 | semi-winter | Hunan, China |
| Xiangzayou553 | XZY553 | semi-winter | Hunan, China |
| Huayouza50 | HZ50 | semi-winter | Hubei, China |
| Huayouza158 | HYZ158 | semi-winter | Hubei, China |
| Dadi199 | DD199 | semi-winter | Hubei, China |
| Zheyou51 | ZY51 | semi-winter | Zhejiang, China |
| Yuyou28 | YY28 | semi-winter | Chongqing, China |
| Qingyou3 | QY3 | semi-winter | Chongqing, China |
| Qingyou1 | QY1 | semi-winter | Chongqing, China |
| Chuangyou81 | CY81 | semi-winter | Sichuang, China |
| Chuangyou36 | CY36 | semi-winter | Sichuang, China |
| Shanyou28 | SY28 | semi-winter | Shaanxi, China |
| Qinyou33 | QY33 | semi-winter | Shaanxi, China |

Table S2 Soil physical properties

|  | | Huanggang | Xiangyang | Wuhan | | |
| --- | --- | --- | --- | --- | --- | --- |
|  |  |  |  | Normal water | Drought | Waterlogged |
| Texture | | Loam | Loam | Loam | | |
| Soil saturated moisture (%) | | 34.9 | 38.5 | 37.22 | | |
| Soil compactness (Pa) | 0-5 cm | 230-930 | 241-738 | 215-738 | 152-773 | 134-713 |
|  | 5-30 cm | 567-2218 | 458-1385 | 712-1986 | 349-1616 | 670-1947 |

Fig. S1 Seedling growth at different sowing depth in Huanggang and Xiangyang

(a, b) Shoot and root length, (c, d) root collar diameter, and (e, f) leaf area in Huanggang and Xiangyang with three sowing depths (1 cm, 3 cm and 5 cm), respectively. Bars represent ± SD of replicates. Small letters represent significant differences at *p* < 0.05 level according to Duncan’s multiple. HG: huanggang, XY: xiangyang, H: huayouza62, Y: huayou2101, Z: zhongshuang11.

Fig. S2 Seedling growth at different sowing depth under different water condition

Root collar diameter and leaf area at the three sowing depths (1, 3 and 5 cm) under (a, b) normal water (SWC = 60–85%), (c-d) drought (SWC = 30–55%) and (e-f) waterlogged (SWC = 90–100%), respectively. Bars represent ± SD of replicates. Small letters represent significant differences at *p* < 0.05 level according to Duncan’s multiple. H: huayouza62, Y: huayou2101, Z: zhongshuang11.

Fig. S3 Seedling emergence growth at different sowing depth under different water condition

Seedling emergence percentage dynamic curve, emergence index (EI) and seedling root to stem ratio at five sowing depths (1, 2, 3, 4 and 5 cm) under (a-c) drought (SWC = 70 ± 5%), (d-f) normal water (SWC = 40 ± 5%) and (g-i) waterlogged (SWC = 100 ± 5%) conditions. Seedling EI of all varieties at five sowing depths was evaluated by curve-fitting analysis under drought conditions, R^2^ represents the correlation of the equation and asterisks indicate significant differences according to t-test (**p* < 0.05). Small letters represent significant differences at *p* < 0.05 level according to Duncan’s multiple.

Fig. S4 Exogenous application of GS improves plant growth under different water conditions with moderately deep sowing

Shoot and root length, root collar diameter and leaf area under (a, b, c) normal water, (d, e, f) drought and (g, h, i) waterlogged conditions at 1 and 3 cm sowing depths after seed priming by GS, respectively. Bars represent ± SD of replicates. Small letters represent significant differences at *p* < 0.05 level according to Duncan’s multiple. ZS11: untreated seed, H_2_O: double distilled water, GS: glucosinolate.
